# Translational chasm and dialogues in clinical neuroscience

**DOI:** 10.1080/19585969.2022.2073566

**Published:** 2022-06-01

**Authors:** Lakshmi N. Yatham, Florence Thibaut

**Affiliations:** a Department of Psychiatry, Director, Institute of Mental Health, University of British Columbia, Vancouver, Canada; b Department of Psychiatry/Addictology, University of Paris Cité, University Hospital Cochin-Tarnier, INSERM U1266, Institute of Psychiatry and Neurosciences, Paris, France

One of the major objectives of the World Federation of Societies of Biological Psychiatry (WFSBP) is to improve the quality of training in all biological psychiatry sciences and to foster scientific research in order to advance the field of biological psychiatry with an ultimate goal to improve patient care. In addition to disseminating advances in biological psychiatry through its biennial Congress, WFSBP publishes World Journal of Biological Psychiatry, which also serves as an important medium for communicating advances in the field.

Translational Research is conceptualised as **‘**Transformation of scientific discoveries arising from laboratory, clinical, or population studies into clinical applications to reduce incidence, morbidity, and mortality of diseases’. However, translational chasm is a significant issue in mental health where there are significant challenges in translating basic science discoveries into clinical arena and even more importantly translating clinical discoveries to real world clinical practice. Indeed, several organisations including National Institute of Health and National Institute of Mental Health declared that reducing the translational gap should be a key priority (Glasgow et al. 2013).

The main focus of the 'Dialogues in Clinical Neuroscience’ (DCNS), an international peer reviewed journal, has been to contribute to reducing the translational gap in mental health by publishing articles that synthesise new advances in clinical neuroscience research that have immediate implications for real world clinical practice. This objective very much aligns with the vision of the WFSBP and we are delighted to share the news that the WFSBP has recently added this new journal to its portfolio of journals. Given the highly clinically relevant nature of the manuscripts that the DCNS has been publishing, it is not surprising that these articles are highly cited contributing to an impact factor of over 5 for the journal. The DCNS under the auspices of WFSBP will continue to be edited by Professor Florence Thibaut with support from a group of international clinician scientists with expertise in translational neuroscience, thus ensuring the adherence to the original vision of the journal. DCNS will be an open access journal with 4 issues in the first year and will include invited state of the art synthesis of clinically relevant neuroscience articles as well as manuscripts that report on original highly relevant clinical research.

True to its vision, this first inaugural issue of the DCNS under the WFSBP, includes articles that address several clinically relevant topics ranging from addressing contemporary topics such as chemical sex to advances in technological tools such as digital phenotyping, blood metabolic profile and graph theory to synthesis of new knowledge on cognitive function in bipolar disorder and gender dysphoria. As well, DCNS will strive to publish research from all parts of the world and the manuscripts included in this first issue reflect that aim. Falsaperla et al. (2021) from Catalania in their systematic review identify the utility of graph theory in aiding the diagnosis of paediatric epilepsy, which has significant clinical implications. Pathological social withdrawal termed ‘Hikikomori’ was originally described in Japan but has since been reported in other countries including non-Asian countries. The pathophysiology of this condition remains unknown. In this issue, Setoyama et al. (2021) from Japan describe a metabolic signature profile, which if replicated has the potential significantly advance our understanding and treatment of this devastating condition. Emil Kraepelin more than 100 years ago described in his text book that patients with dementia praecox (now called schizophrenia) experience cognitive deterioration but not those with manic depressive psychosis (i.e., bipolar disorder) who predominantly experience mood changes with periods of exacerbation and remission. Kraepelin was a master clinician and most of his observations on major psychiatric illnesses were proven correct; the only exception being cognitive impairment as the research over the last two decades has shown that about a half to two thirds of patients with bipolar disorder do demonstrate some cognitive impairment. Keramatian et al. (2021) from Canada provide a state of the art summary of what is known about cognition in bipolar disorder to date and the implications of this research to real world clinical practice. Chemical sex is a new and evolving topic and the substances used for promoting sex may have psychotropic effects. The reported prevalence of over 20% of chemical sex in university students (Malandain et al. 2021) is concerning and suggest that more research and knowledge dissemination about this topic to clinicians is urgently needed. Crocq (2021) describes the evolution of the concept of gender dysphoria from Ancient Greek and Roman times to the current DSM.5 and ICD-11, providing critically important insights for clinicians. Finally, Mouchabac et al. (2021) describe the potential promise and utility of digital phenotyping which combined with advances in machine learning could aid in clinical decisions such as diagnosis and treatment of psychiatric illnesses.

We hope that the DCNS will continue to play an important role in bringing highly clinically relevant topics to the clinicians in order to reduce the translational gap in psychiatry.
